# Case report: Treatment of long COVID with a SARS-CoV-2 antiviral and IL-6 blockade in a patient with rheumatoid arthritis and SARS-CoV-2 antigen persistence

**DOI:** 10.3389/fmed.2022.1003103

**Published:** 2022-09-23

**Authors:** Lavanya Visvabharathy, Zachary S. Orban, Igor J. Koralnik

**Affiliations:** Ken and Ruth Davee Department of Neurology, Feinberg School of Medicine, Northwestern University, Chicago, IL, United States

**Keywords:** long COVID, nirmatrelvir/ritonavir, tocilizumab, autoimmunity, case report

## Abstract

**Introduction:**

Long COVID, or post-acute sequelae of SARS-CoV-2 infection (PASC) in ∼30% of all infected individuals. Here, we present a case of PASC in a patient with rheumatoid arthritis characterized by viral persistence in the nasopharynx for 6 months after acute infection. We demonstrate transient disappearance of antigen persistence and decreased antiviral and autoimmune T cell responses after nirmatrelvir/ritonavir and tocilizumab treatment.

**Case presentation:**

A 37-year-old female with a 7-year history of rheumatoid arthritis enrolled in a COVID-19 research study was found to continuously test SARS-CoV-2 antigen positive in the nasopharynx for 6 months after acute infection. She simultaneously presented with new-onset PASC symptoms including chronic occipital headache and periods of intense fatigue 8 weeks after acute infection. The patient was prescribed nirmatrelvir/ritonavir to treat SARS-CoV-2 persistence at 3.5 months post-acute infection and observed a reduction in PASC symptoms 3 weeks after completing antiviral treatment. After resurgence of PASC symptoms, she stopped treatment with tocilizumab for rheumatoid arthritis to attempt complete SARS-CoV-2 viral clearance. The severity of the patient’s PASC symptoms subsequently increased, and she developed new-onset brain fog in addition to previous symptoms, which resolved after resumption of tocilizumab treatment. Assessment of adaptive immune responses demonstrated that nirmatrelvir/ritonavir and tocilizumab treatment decreased antiviral and autoreactive T cell activation. After resuming tocilizumab treatment, the patient’s PASC symptoms were significantly reduced, but nasopharyngeal antigen positivity remained.

**Conclusion:**

These data suggest that nirmatrelvir/ritonavir should be considered in the treatment of PASC in patients who have SARS-CoV-2 antigen persistence, though care must be taken to monitor the patient for symptom resurgence or viral reactivation. In addition, the IL-6 inhibitor tocilizumab may ameliorate PASC symptoms in patients with persistent headache, fatigue, and brain fog.

## Introduction

SARS-CoV-2 is a (+)-strand RNA β-coronavirus first identified in December, 2019 and is the causative agent of COVID-19. There have been more than 560 million cases and 6.3 million deaths globally attributable to the COVID-19 pandemic (1). Although highly effective vaccines are now used to prevent severe disease and death from SARS-CoV-2, long-term sequelae after infection have become an urgent medical concern (2, 3). The rapid emergence of variants with enhanced virulence profiles and increased ability to evade vaccine-elicited immunity make it crucial to find alternative treatment options for COVID-related sequelae (4).

Post-acute sequelae of SARS-CoV-2 infection (PASC), or “long COVID,” includes symptoms persisting for more than 4 weeks after acute infection and affects an estimated 30% of people infected with SARS-CoV-2 (5). Neuro-PASC is clinically defined as new neurologic or neurocognitive symptoms persisting for more than 4 weeks after disease onset and is often not concomitant with diagnosis of acute infection (6, 7). Currently, there are only symptomatic treatment options for Neuro-PASC (8), demonstrating the urgent need for new therapeutic approaches that address the underlying cause(s).

We describe a unique case of Neuro-PASC in a patient with a 7-year history of rheumatoid arthritis (RA). The patient tested continuously positive by SARS-CoV-2 by FlowFlex rapid antigen test for more than 6 months after acute infection. Treatment with nirmatrelvir/ritonavir and modifying treatment with tocilizumab decreased or eliminated PASC symptoms as well as antiviral and autoreactive T cell responses.

## Case presentation

**FIGURE 1:**
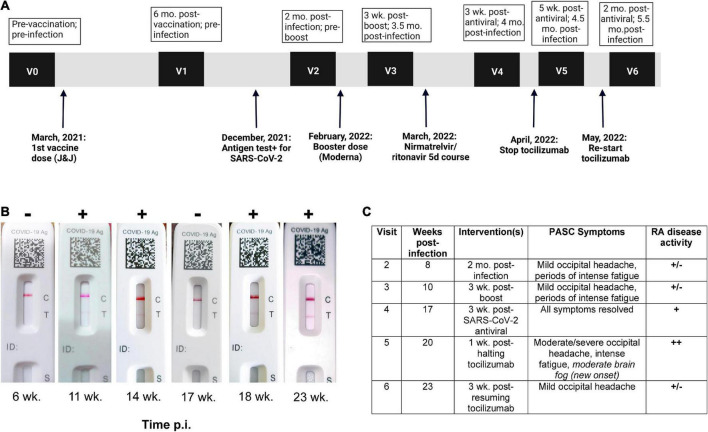
Post-acute sequelae of SARS-CoV-2 infection (PASC) patient exhibits persistent SARS-CoV-2 antigen positivity in nasopharynx. **(A)** Study visit timeline, including vaccination, infection, and intervention dates. **(B)** FlowFlex™ SARS-CoV-2 antigen test results over time. Time p.i., weeks post-infection. **(C)** PASC symptoms vs. rheumatoid arthritis symptoms.

A 37-year-old South Asian woman on bi-weekly 162 mg/ml tocilizumab injections for RA was enrolled in a Neuro-PASC research study at Northwestern University in Chicago (demographics in Figure 1A; study design in Figure 1B). She was acutely symptomatic with new onset severe fatigue, occipital headache, and loss of appetite. She tested SARS-CoV-2^+^ by nasopharyngeal rapid antigen test in December, 2021. She experienced persistent headache and fatigue for >6 weeks after infection. The patient tested RT-PCR^–^ for SARS-CoV-2 at 14 days post-infection and multiple times thereafter but continued to test intermittently antigen^+^ for 14 weeks post-infection despite no overt exposure to SARS-CoV-2 infected individuals. The patient lived alone, did not leave her residence without a surgical-grade N95 mask, and never removed the mask in public. She was subsequently prescribed a 5-day course of nirmeltravir/ritonavir 300/100 mg twice daily, a SARS-CoV-2-specific antiviral, on the basis of her continued positive antigen tests. Patient was in compliance with all prescribed treatment courses. Initially, all PASC symptoms resolved, and the patient tested antigen^–^ 3 weeks after completion of nirmeltravir/ritonavir, but PASC symptoms and antigen positivity subsequently reappeared at 4 weeks (Figures 1B,C). The patient halted tocilizumab therapy for RA upon her rheumatologist’s recommendation for 10 days in an attempt to fully clear the virus, during which time she developed more severe PASC symptoms and the appearance of new-onset cognitive impairment (brain fog). She resumed tocilizumab and within 3 weeks, her fatigue and brain fog had resolved while the occipital headache decreased in severity (Figure 1C).

**FIGURE 2:**
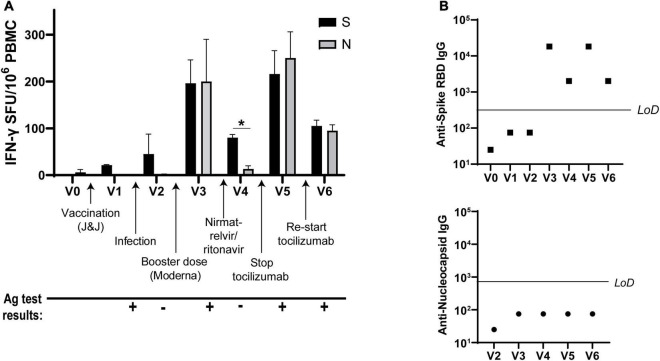
IFN-*γ* T cell responses to SARS-CoV-2 vary over time. **(A)** IFN-*γ* production from SARS-CoV-2 Spike- and Nucleocapsid-specific T cells at each visit as determined by ELISPOT. **(B)** Spike receptor-binding domain (RBD)-specific IgG titers (top) and Nucleocapsid-specific IgG (bottom) at each visit. LoD, limit of detection. All ELISPOT data in panel **(A)** from duplicate wells. Data representative of 2 individual experiments, *p < 0.05 by Student’s *t*-test.

Analysis of antiviral T cell responses by IFN-*γ* ELISPOT showed that Spike-specific T cell activation was induced by vaccination and infection, as expected after receiving Spike protein-based vaccines. However, the patient’s non-Spike responses (to Nucleocapsid) were enhanced as well after receiving the Moderna vaccine booster dose. Nirmatrelvir/ritonavir treatment resulted in the retention of Spike- but not Nucleocapsid-specific T cell responses, while halting tocilizumab correlated with elevated T cell responses against both proteins. Resumption of tocilizumab subsequently decreased T cell responses to both viral antigens (Figure 2A). Antibody titers against Spike receptor-binding domain (RBD) followed similar kinetics (Figure 2B, top), while the patient never mounted an antibody response against Nucleocapsid (Figure 2B, bottom).

**FIGURE 3:**
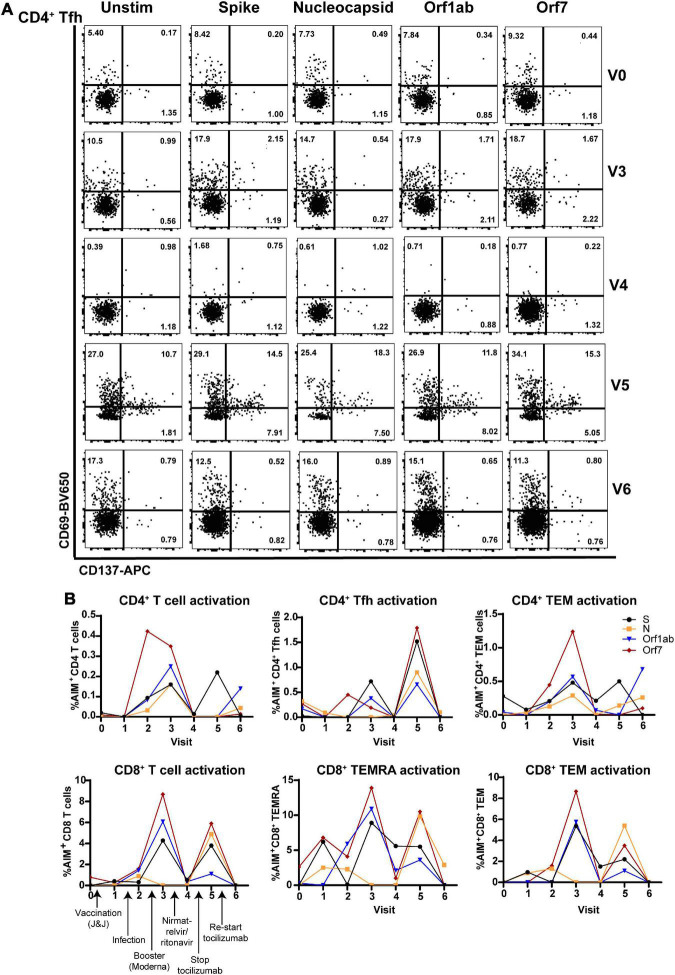
Virus-specific CD4^+^ and CD8^+^ T cell subset activation correlates with antiviral and tocilizumab treatment. **(A)** Flow cytometry showing elevated virus-specific CD4^+^ T helper cell (Tfh) cell activation after vaccine booster dose (V3; 2nd row) and stopping tocilizumab treatment (V5; 4th row). **(B)** Total CD4^+^ T cells (left), CD4^+^ T helper cells (Tfh, middle), and CD4^+^ memory T cells (TEM, right) have enhanced reactivity to SARS-CoV-2 structural (S, N) and non-structural (Orf1ab, Orf7) peptides after vaccine booster (V3) and stopping tocilizumab (V5), but low reactivity after nirmatrelvir/ritonavir treatment (V4) and resuming tocilizumab therapy (V6). **(B)** Total CD8^+^ T cells (right) and CD8^+^ memory T cell subsets (CD8^+^ TEMRA, TEM; middle, right) show increased activation after vaccine boost (V3) and stopping tocilizumab (V5), but low reactivity after nirmatrelvir/ritonavir treatment (V4) and resuming tocilizumab (V5). Data combined from 3 independent experiments.

Flow cytometric analysis of (antibodies used in Supplementary Table 1) CD4^+^ T follicular helper cells (Tfh; involved in T cell help for antibody production) showed that antiviral T cell activation was highest at V3 (post-infection, post-boost) and V5 (post-infection, -boost, -nirmatrelvir/ritonavir, and halting tocilizumab treatment). Tfh activation determined by the activation-induced marker assay (AIM) (9) was lowest 3 weeks after nirmatrelvir/ritonavir treatment and resumption of tocilizumab (Figure 3A). Similarly, total CD4^+^ and CD8^+^ T cells, CD4^+^ and CD8^+^ T effector memory (TEM) cells, and CD8^+^ TEM cells re-expressing CD45RA (CD8^+^ TEMRA; terminally differentiated and highly cytotoxic T cells) exhibited maximal SARS-CoV-2-specific activation to Spike, Nucleocapsid, Orf1ab, and Orf7 antigens at V3 and V5, with limited activation at V4 and V6 (Figure 3B).

**FIGURE 4:**
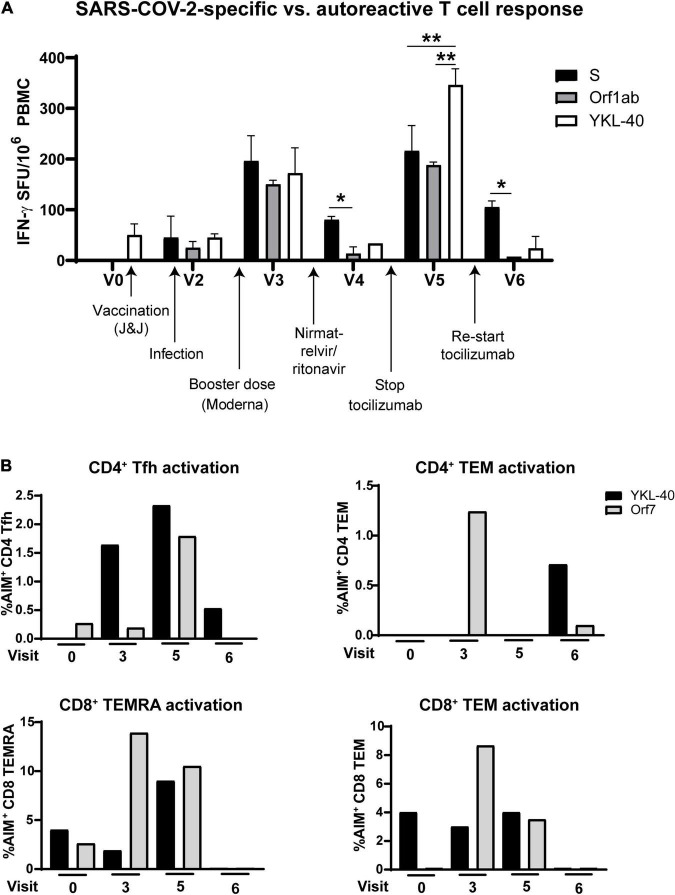
Autoreactive T cell responses oscillate in coordination with SARS-CoV-2-specific responses after infection. **(A)** T cell production of IFN-*γ* after stimulation with rheumatoid arthritis-associated cartilage autoantigen YKL-40 compared with Spike- and Orf1ab-specific activation. **(B)** Activation of T cell subsets over the course of the study after stimulation with the rheumatoid arthritis-associated antigen YKL-40 or SARS-CoV-2 Orf7 peptides. Stopping tocilizumab resulted in increased virus-specific and autoreactive T cell reactivity, while resuming tocilizumab suppressed autoreactivity in most T cell subsets. Data representative of 2 individual experiments, *p < 0.05, **p < 0.01 by one-way ANOVA with Tukey’s post-test.

Antiviral and autoreactive T cell responses demonstrated a parallel oscillation over time. Comparison of IFN-*γ* production from T cells in response to Spike and Orf1ab vs. the RA-associated cartilage antigen YKL-40 showed the highest activation at V3 and V5, and the lowest activation at V4 after nirmatrelvir/ritonavir and V6 after resuming tocilizumab treatment (Figure 4A). Flow cytometry revealed similar activation patterns in T cell memory and Tfh cell subsets (Figure 4B). No other clinical diagnostic testing was performed on the patient.

## Discussion and conclusion

COVID-19 is increasingly being recognized as a multi-organ disease with long-term sequelae associated with neurological dysfunction. PASC has been reported in up to 30% of those with mild disease who do not require hospitalization (10, 11). Long-term sequelae after coronavirus infections can persist for years (12); therefore, individual case reports can inform us on how PASC symptoms may be impacted by available treatment options.

This case study described a Neuro-PASC patient presenting with long-term nasopharyngeal viral shedding as determined by SARS-CoV-2 antigen tests. Persistent viral colonization has been described previously both in the nasopharynx and extra-respiratory sites (13, 14) and is associated with being immunocompromised (15), though it is unknown whether viral persistence is more common in PASC patients than in healthy COVID convalescents. In this case, the patient was on immunosuppressive therapy with tocilizumab for pre-existing RA when she contracted SARS-CoV-2, which may have contributed to viral persistence over 6 months. However, tocilizumab may also decrease the severity of acute SARS-CoV-2 infection in hospitalized patients (16). The patient’s mild acute symptoms combined with an escalation in Neuro-PASC symptom severity after stopping tocilizumab, as well as their resolution after resuming treatment suggests that IL-6 blockade should be studied further as a potential therapeutic intervention for Neuro-PASC.

Nirmatrelvir/ritonavir (Paxlovid) treatment is indicated within the first 72 h of a confirmed SARS-CoV-2 infection diagnosis to limit disease progression and decrease symptom severity (17). Though not indicated for the treatment of PASC, the treating physician felt that her prolonged nasopharyngeal antigen positivity warranted the treatment. Indeed, the patient’s PASC symptoms fully resolved 3 weeks after completing antiviral treatment, which was corroborated by testing antigen^–^ (Figure 1C) and having decreased non-Spike adaptive immune responses (Figures 2, 3). Her symptoms and antigen positivity resumed 4 weeks post-nirmatrelvir/ritonavir treatment along with enhanced T cell and antibody responses to SARS-CoV-2 (Figures 2, 3), suggestive of viral reactivation after antiviral treatment. SARS-CoV-2 viral rebound has been reported after a 5-day course of nirmatrelvir/ritonavir during acute infection (18, 19). A similar mechanism could be at play in the case of Neuro-PASC given evidence of a persistent SARS-CoV-2 infection.

Increased IL-6 has been associated with PASC in multiple studies. Elevated serum IL-6 was found in the majority of PASC patients compared with asymptomatic convalescent controls 8 months post-infection (20). There is speculation that IL-6 dysfunction may underlie persistent neuropsychiatric symptoms of PASC (21), and IL-6-producing B effector cells were elevated relative to IL-10-producing B regulatory cells in severe COVID-19 patients (22). Finally, a large cohort study of PASC patients assessed at 5 months post-infection found increased plasma IL-6 levels in patients with severe or moderate symptoms with cognitive impairment (23). Our own group has shown that Neuro-PASC patients have increased CD8^+^ T cell production of IL-6 following stimulation with SARS-CoV-2 antigens compared with healthy COVID convalescents (24). Combined with the data in this case report, these studies provide a compelling argument for investigating the efficacy of IL-6 blockade with tocilizumab in treatment of PASC.

A confounding factor in characterizing the current patient’s PASC symptoms and accompanying immune responses was her pre-existing RA. RA has been associated with an increased susceptibility to infections even in the absence of immunosuppressive therapy (25). There are also significant positive correlations between susceptibility to PASC and pre-existing autoimmune disease diagnoses (2). In this case, the patient’s autoreactive T cell response to the RA-associated cartilage antigen YKL-40 (26) fluctuated depending on PASC symptom severity and RA disease activity (Figure 1C) as well as tocilizumab treatment (Figure 4). This suggests that tocilizumab may be used for management of rheumatic disease as well as Neuro-PASC symptoms. This case report highlights the need for further research into the contribution of autoimmunity to Neuro-PASC in addition to exploring the use of tocilizumab in Neuro-PASC treatment.

Currently, there are only symptomatic treatments for Neuro-PASC (8). We present the first case of a Neuro-PASC patient whose symptoms and antiviral adaptive immune responses were reduced by a 5-day course of nirmatrelvir-ritonavir. Additionally, we raise the possibility that tocilizumab should be further studied as a therapeutic intervention for Neuro-PASC. PASC impacts millions of people worldwide, and its incidence is only modestly diminished by vaccination (27, 28). Urgent research is needed to study the role of existing treatments to ameliorate the devastating impacts of PASC.

## Limitations

The first author performed all experiments and analyses, and thus the study could not be blinded. We also note that this is one patient’s experience and thus should not be used to generalize to larger patient populations without further clinical trials.

## Author disclosure

The first author is the patient described in the study and gave full consent to use the data in this manuscript.

## Data availability statement

The raw data supporting the conclusions of this article will be made available by the authors upon request, without undue reservation.

## Ethics statement

The studies involving human participants were reviewed and approved by Northwestern University Institutional Review Board. The patients/participants provided their written informed consent to participate in this study. IRB study number STU00212583. Written informed consent was obtained from the individual(s) for the publication of any potentially identifiable images or data included in this article.

## Author contributions

LV: conceptualization. LV and ZO: investigation and formal analysis. LV and IK: resources, data curation, supervision, project administration, and funding acquisition. LV: writing with feedback from all authors. All authors contributed to the article and approved the submitted version.

## References

[1] Johns Hopkins Coronavirus Resource Center. *Cumulative Worldwide Covid-19 Cases.* Baltimore, MD: Johns Hopkins University of Medicine (2021).

[2] Graham EL Clark JR Orban ZS Lim PH Szymanski AL Taylor C Persistent neurologic symptoms and cognitive dysfunction in non-hospitalized Covid-19 “long haulers”. *Ann Clin Transl Neurol.* (2021) 8:1073–85. 10.1002/acn3.51350 33755344 PMC8108421

[3] Higgins V Sohaei D Diamandis EP Prassas I. COVID-19: From an acute to chronic disease? Potential long-term health consequences. *Crit Rev Clin Lab Sci.* (2020). 58, 297–310. 10.1080/10408363.2020.1860895 33347790

[4] Newman J Thakur N Peacock TP Bialy D Elrefaey AME Bogaardt C Neutralizing antibody activity against 21 SARS-CoV-2 variants in older adults vaccinated with BNT162b2. *Nat Microbiol.* (2022) 7:1180–8. 10.1038/s41564-022-01163-3 35836002 PMC9352594

[5] Ladds E Rushforth A Wieringa S Taylor S Rayner C Husain L Persistent symptoms after Covid-19: qualitative study of 114 “long Covid” patients and draft quality principles for services. *BMC Health Serv Res.* (2020) 20:1144. 10.1186/s12913-020-06001-y 33342437 PMC7750006

[6] Moghimi N Di Napoli M Biller J Siegler JE Shekhar R McCullough LD The Neurological manifestations of post-acute sequelae of SARS-CoV-2 infection. *Curr Neurol Neurosci Rep.* (2021) 21:44. 10.1007/s11910-021-01130-1 34181102 PMC8237541

[7] Nalbandian A Sehgal K Gupta A Madhavan MV McGroder C Stevens JS Post-acute COVID-19 syndrome. *Nat Med.* (2021) 27:601–15. 10.1038/s41591-021-01283-z 33753937 PMC8893149

[8] Graham EL Koralnik IJ Liotta EM. Therapeutic Approaches to the Neurologic Manifestations of COVID-19. *Neurotherapeutics.* (2022) [Epub ahead of print]. 10.1007/s13311-022-01267-y 35861926 PMC9302225

[9] Havenar-Daughton C Reiss SM Carnathan DG Wu JE Kendric K Torrents de la Pena A Cytokine-independent detection of antigen-specific germinal center t follicular helper cells in immunized nonhuman primates using a live cell activation-induced marker technique. *J Immunol.* (2016) 197:994–1002. 10.4049/jimmunol.1600320 27335502 PMC4955744

[10] Hirschtick JL Titus AR Slocum E Power LE Hirschtick RE Elliott MR Population-based estimates of post-acute sequelae of SARS-CoV-2 infection (PASC) prevalence and characteristics. *Clin Infect Dis.* (2021) 73:2055–64. 10.1093/cid/ciab408 34007978 PMC8240848

[11] Havervall S Rosell A Phillipson M Mangsbo SM Nilsson P Hober S Symptoms and functional impairment assessed 8 months after mild COVID-19 among health care workers. *JAMA.* (2021) 325:2015–6. 10.1001/jama.2021.5612 33825846 PMC8027932

[12] Ahmed H Patel K Greenwood DC Halpin S Lewthwaite P Salawu A Long-term clinical outcomes in survivors of severe acute respiratory syndrome and middle east respiratory syndrome coronavirus outbreaks after hospitalisation or ICU admission: a systematic review and meta-analysis. *J Rehabil Med.* (2020) 52:jrm00063. 32449782 10.2340/16501977-2694

[13] Ferrari A Trevenzoli M Sasset L Di Liso E Tavian T Rossi L Prolonged SARS-CoV-2-RNA detection from nasopharyngeal swabs in an oncologic patient: What impact on cancer treatment? *Curr Oncol.* (2021) 28:847–52. 10.3390/curroncol28010083 33567626 PMC7985795

[14] Natarajan A Zlitni S Brooks EF Vance SE Dahlen A Hedlin H Gastrointestinal symptoms and fecal shedding of SARS-CoV-2 RNA suggest prolonged gastrointestinal infection. *Med.* (2022) 3:371–387.e9. 10.1016/j.medj.2022.04.001 35434682 PMC9005383

[15] Thornton CS Huntley K Berenger BM Bristow M Evans DH Fonseca K Prolonged SARS-CoV-2 infection following rituximab treatment: clinical course and response to therapeutic interventions correlated with quantitative viral cultures and cycle threshold values. *Antimicrob Resist Infect Control.* (2022) 11:28. 10.1186/s13756-022-01067-1 35123573 PMC8817557

[16] Snow TAC Saleem N Ambler G Nastouli E Singer M Arulkumaran N. Tocilizumab in COVID-19: a meta-analysis, trial sequential analysis, and meta-regression of randomized-controlled trials. *Intensive Care Med.* (2021) 47:641–52. 10.1007/s00134-021-06416-z 34019122 PMC8139226

[17] Wen W Chen C Tang J Wang C Zhou M Cheng Y Efficacy and safety of three new oral antiviral treatment (molnupiravir, fluvoxamine and Paxlovid) for COVID-19a meta-analysis. *Ann Med.* (2022) 54:516–23. 10.1080/07853890.2022.2034936 35118917 PMC8820829

[18] Boucau J Uddin R Marino C Regan J Flynn JP Choudhary MC Characterization of virologic rebound following nirmatrelvir-ritonavir treatment for COVID-19. *Clin Infect Dis.* (2022) [Epub ahead of print]. 10.1093/cid/ciac512 35737946 PMC9384370

[19] Wang L Berger NA Davis PB Kaelber DC Volkow ND Xu R. COVID-19 rebound after Paxlovid and Molnupiravir during January-June 2022. *medRxiv* [Preprint]. (2022) 10.1101/2022.06.21.22276724 35794889 PMC9258292

[20] Phetsouphanh C Darley DR Wilson DB Howe A Munier CML Patel SK Immunological dysfunction persists for 8 months following initial mild-to-moderate SARS-CoV-2 infection. *Nat Immunol.* (2022) 23:210–6. 10.1038/s41590-021-01113-x 35027728

[21] Kappelmann N Dantzer R Khandaker GM. Interleukin-6 as potential mediator of long-term neuropsychiatric symptoms of COVID-19. *Psychoneuroendocrinology.* (2021) 131:105295. 10.1016/j.psyneuen.2021.105295 34119855 PMC8172271

[22] Shuwa HA Shaw TN Knight SB Wemyss K McClure FA Pearmain L Alterations in T and B cell function persist in convalescent COVID-19 patients. *Med.* (2021) 2:720–735.e4. 10.1016/j.medj.2021.03.013 33821250 PMC8011689

[23] Evans RA McAuley H Harrison EM Shikotra A Singapuri A Sereno M. Clinical characteristics with inflammation profiling of long COVID and association with 1-year recovery following hospitalisation in the UK: a prospective observational study. *Lancet Respir Med.* (2022) 10:761–75. 10.1016/S2213-2600(22)00127-8 35472304 PMC9034855

[24] Visvabharathy L Hanson B Orban Z Lim PH Palacio NM Jain R Neuro-COVID long-haulers exhibit broad dysfunction in T cell memory generation and responses to vaccination. *medRxiv* [Preprint]. (2021) 10.1101/2021.08.08.21261763 34401886 PMC8366804

[25] Listing J Gerhold K Zink A. The risk of infections associated with rheumatoid arthritis, with its comorbidity and treatment. *Rheumatology.* (2013) 52:53–61. 10.1093/rheumatology/kes305 23192911

[26] Sekine T Masuko-Hongo K Matsui T Asahara H Takigawa M Nishioka K Recognition of YKL-39, a human cartilage related protein, as a target antigen in patients with rheumatoid arthritis. *Ann Rheum Dis.* (2001) 60:49–54. 10.1136/ard.60.1.49 11114282 PMC1753367

[27] Al-Aly Z Bowe B Xie Y. Long COVID after breakthrough SARS-CoV-2 infection. *Nat Med.* (2022) 28:1461–7. 10.1038/s41591-022-01840-0 35614233 PMC9307472

[28] Azzolini E Levi R Sarti R Pozzi C Mollura M Mantovani A Association between BNT162b2 vaccination and long COVID after infections not requiring hospitalization in health care workers. *JAMA.* (2022) 328:676. 10.1001/jama.2022.11691 35796131 PMC9250078

