# Supplementary material for: Case report: Treatment of long COVID with a SARS-CoV-2 antiviral and IL-6 blockade in a patient with rheumatoid arthritis and SARS-CoV-2 antigen persistence
Source: Front Med (Lausanne). 2022 Sep 23;9:1003103. doi: 10.3389/fmed.2022.1003103 (PMC9537824; doi:10.3389/fmed.2022.1003103)
Supplement: Supplementary file 1 [file Data_Sheet_1.pdf]

## Supplemental Methods

*Ethics Statement*

This study was approved by the Northwestern University Institutional Review Board (Koralnik Lab, IRB STU00212583). Informed consent was obtained from participant. Samples were de-identified before banking.

*Study participant*

We enrolled the individual patient as a part of our Neuro-COVID-19 immunology research study. Patient was confirmed to be SARS-CoV-2 antigen$^+$ prior to enrollment and to have lingering self-reported Neuro-PASC symptoms. Subject remained living throughout the period of observation. Heparinized blood samples were collected at 7 individual time points. Demographic information contained in Fig. 1A.

*PBMC and plasma collection*

30mL of venous blood from patient was collected in blood collection tubes containing sodium heparin from BD Biosciences. Whole blood was layered on top of 15mL of Histopaque 1077 (Sigma-Aldrich) in 50mL Leucosep blood separation tubes (Greiner Bio-One) and spun at 1000g x 18min at RT. Plasma was collected and stored at -80°C. The PBMC layer was collected and washed 2x in sterile PBS before red blood cell lysis with ACK buffer (Quality Biologicals). PBMCs were used in assays either immediately or frozen down for use in the near term.

*Peptide antigens*

All S and N peptide arrays used in ELISPOT and flow cytometry studies were obtained from BEI Resources, NIAID, NIH: Peptide Array, SARS-Related Coronavirus 2 Spike (S) Protein; NR-52402, Nucleocapsid (N) Protein, NR-52404. Orf1ab and Orf7 peptides were obtained from JPT (Berlin, Germany). YKL-40 peptides were obtained from GenScript (Piscataway, NJ). S, N, and YKL-40 peptides were 13-15-mers overlapping by 10-11 amino acids. Orf1ab and Orf7 peptides were predicted $CD8^+$ T cell epitopes as defined in MIRA assays and TCR sequencing [24] by Adaptive Biotechnologies (Seattle, WA).

*IgG Spike RBD and Nucleocapsid ELISA*

Antigen-specific total antibody titers were measured by ELISA as described previously [25]. In brief, 96-well flat-bottom MaxiSorp plates (Thermo Scientific) were coated with 1 µg/ml of Spike RBD for 48 hr at 4°C. Plates were washed three times with wash buffer. Blocking was performed with blocking solution for 4 hr at room temperature. 6 µl of sera was added to 144 µl of blocking solution in the first column of the plate, 1:3 serial dilutions were performed until row 12 for each sample, and plates were incubated for 60 min at room temperature. Plates were washed three times with wash buffer followed by addition of secondary antibody conjugated to horseradish peroxidase, goat anti-human IgG (H + L) (Jackson ImmunoResearch) diluted in blocking solution (1:1000) and 100 µl/well was added and incubated for 60 min at room temperature. After washing plates three times with wash buffer, 100 µl/well of Sure Blue substrate (SeraCare) was added for 1 min. Reaction was stopped using 100 µl/well of KPL TMB Stop Solution (SeraCare). Absorbance was measured at 450 nm using a Spectramax Plus 384 (Molecular Devices). SARS-CoV-2 RBD and N proteins used for ELISA were produced at the Northwestern Recombinant Protein Production Core by Dr. Sergii Pshenychnyi using plasmids that were produced under HHSN272201400008C and obtained from BEI Resources, NIAID, NIH: Vector

pCAGGS containing the SARS-related coronavirus 2, Wuhan-Hu-1 spike glycoprotein gene (soluble, stabilized), NR-52394 and receptor binding domain (RBD), NR-52309, nucleocapsid gene NR-53507.

*Cell stimulation and IFN-γ/IL-2 ELISPOT*

Multiscreen-IP plates (Millipore-Sigma) were coated overnight at 4°C with 2μg/mL anti-IFN-γ (clone 1-D1K, Mabtech), washed with sterile PBS, and blocked with complete RPMI-10% FBS. PBMC isolated from Neuro-PASC, COVID convalescent, and healthy control subjects were used either freshly isolated or after thawing and resting overnight in media containing 10ng/μL recombinant human IL-15 (Peprotech) at 37°C, 5% $CO_2$. Cells were then plated at a concentration of $2.5x10^5$ cells/well in 100μL of media and stimulated with the indicated antigen mixtures from SARS-CoV-2 at a concentration of 2μg/mL in complete RPMI medium containing 5% human AB serum (Sigma-Aldrich) and 5ng/mL IL-15. Plates were incubated at 37°C, 5% $CO_2$ for 20h and washed 5x with $dH_2O$ and PBS-0.05% Tween-20 (PBS-T). 2μg/mL biotinylated IFN-γ (clone 7-B6-1, Mabtech) or 5μg/mL IL-2 (clone MT8G10, Mabtech) diluted in PBS-10% FBS (PBS-F) was added to the respective wells and plates were incubated for 1.5h at RT. Plates were subsequently incubated for 40 minutes at RT in streptavidin-alkaline phosphatase in PBS-F (Jackson ImmunoResearch) was added after washing plates 5x in PBS-T. ELISPOT plates were developed using an Alkaline Phosphatase Conjugate Substrate Kit according to manufacturer's instructions (Bio-Rad Laboratories, Carlsbad, CA). IFN-γ-producing cells were quantified using an ImmunoSpot reader (Cellular Technologies, Ltd., Shaker Heights, OH).

*Antibodies and Flow Cytometry*

Fresh or frozen PBMCs isolated from the indicated patient groups were stimulated with antigen mixtures as above for 20-22h at 37°C, 5% $CO_2$. Cells were washed with PBS-1% BSA after incubation and incubated with the indicated antibodies for surface phenotyping by AIM assay (antibodies used described in Supplemental Table 1). Cells from each subject were left unstimulated in medium containing 5ng/mL IL-15 ("background") or stimulated in the presence of the indicated antigens. Surface staining was done in the dark at 4°C for 30 minutes. Flow cytometry was conducted on $2\text{-}5x10^5$ cells per condition. Data was acquired on a BD FACSymphony Spectral analyzer and analyzed using FlowJo v10 (BD Biosciences).

*Quantification and Statistical Analysis*

Statistical tests to determine significance are described in figure legends and conducted in Prism (GraphPad). All error bars on figures represent values ± SEM.

**Supplemental Table 1: Antibodies used in study**

| Antibody | Source | Clone | Identifier (Cat. No.) |
|---|---|---|---|
| CD3-BUV395 | BD Biosciences | SK7 | 564001 |
| CD38-BUV496 | BD Biosciences | HIT2 | 612947 |
| CD137-APC | Biolegend | 4-1BB | 309810 |
| CCR7-BUV737 | BD Biosciences | 3D12 | 741786 |
| CD27-BUV805 | BD Biosciences | L125 | 748704 |
| CD8-V500 | BD Biosciences | SK1 | 561618 |
| CD45RA-BV570 | Biolegend | HI100 | 304132 |
| CD45RO-BV605 | Biolegend | UCHL1 | 304238 |
| CD4-BV711 | BD Biosciences | SK3 | 563028 |
| HLA-DR-BV480 | BD Biosciences | G46-6 | 566113 |
| CXCR5-PE-Dazzle 594 | Biolegend | J252D4 | 356928 |
| ICOS-AF700 | Biolegend | C398.4A | 313528 |
| CD69-BV650 | Biolegend | FN50 | 310934 |
| CD134-APC-Fire 750 | Biolegend | Ber-ACT35 | 350032 |
| CD25-BV785 | Biolegend | BC96 | 302638 |
| CXCR3-PE | Biolegend | G025H7 | 353706 |
| PD-1-BV421 | BD Biosciences | EH12.1 | 562516 |
